# Clinical utility of the at-risk for psychosis state beyond transition: A multidimensional network analysis

**DOI:** 10.1007/s00787-024-02491-x

**Published:** 2024-06-19

**Authors:** Tommaso Boldrini, Gabriele Lo Buglio, Erika Cerasti, Maria Pontillo, Laura Muzi, Silvia Salcuni, Andrea Polari, Stefano Vicari, Vittorio Lingiardi, Marco Solmi

**Affiliations:** 1 Department of Developmental Psychology and Socialization, University of Padova, Padua, Italy; 2 Department of Dynamic and Clinical Psychology, and Health Studies, Faculty of Medicine and Psychology, Sapienza University of Rome, Rome, Italy; 3 Child Psychiatry Unit, Department of Neuroscience, Bambino Gesù Children’s Hospital, IRCCS, Rome, Italy; 4 Department of Philosophy, Social Sciences, Humanities and Education, University of Perugia, Perugia, Italy; 5 Orygen Specialist Programs, Melbourne, Australia; 6 Centre for Youth Mental Health, University of Melbourne, Melbourne, Australia; 7 Department of Life Science and Public Health, Catholic University of the Sacred Heart, Rome, Italy; 8 School of Epidemiology and Public Health, Faculty of Medicine, University of Ottawa, Ottawa, ON Canada; 9 Department of Psychiatry, University of Ottawa, Ottawa, ON Canada; 10 The Champlain First Episode Psychosis Program, Department of Mental Health, The Ottawa Hospital, Ottawa, ON Canada; 11 Ottawa Hospital Research Institute (OHRI) Clinical Epidemiology Program University of Ottawa, Ottawa, ON Canada; 12 Department of Child and Adolescent Psychiatry, Charité Universitätsmedizin, Berlin, Germany; 13 Department of Psychology and Educational Science, Pegaso Telematic University, Naples, Italy

**Keywords:** Clinical high-risk for psychosis (CHR-P), Network analysis, Comorbidity, Positive symptoms, Depression, Anxiety

## Abstract

To be relevant to healthcare systems, the clinical high risk for psychosis (CHR-P) concept should denote a specific (i.e., unique) clinical population and provide useful information to guide the choice of intervention. The current study applied network analyses to examine the clinical specificities of CHR-P youths compared to general help-seekers and non–CHR-P youth. 146 CHR-P (mean age = 14.32 years) and 103 non–CHR-P (mean age = 12.58 years) help-seeking youth were recruited from a neuropsychiatric unit and assessed using the Structured Interview for Psychosis-Risk Syndromes, Children’s Depression Inventory, Multidimensional Anxiety Scale for Children, Global Functioning: Social, Global Functioning: Role, and Wechsler Intelligence Scale for Children/Wechsler Adult Intelligence Scale. The first network structure comprised the entire help-seeking sample (i.e., help-seekers network), the second only CHR-P patients (i.e., CHR-P network), and the third only non–CHR-P patients (i.e., non–CHR-P network). In the help-seekers network, each variable presented at least one edge. In the CHR-P network, two isolated “archipelagos of symptoms” were identified: (a) a subgraph including functioning, anxiety, depressive, negative, disorganization, and general symptoms; and (b) a subgraph including positive symptoms and the intelligence quotient. In the non–CHR-P network, positive symptoms were negatively connected to functioning, disorganization, and negative symptoms. Positive symptoms were less connected in the CHR-P network, indicating a need for specific interventions alongside those treating comorbid disorders. The findings suggest specific clinical characteristics of CHR-P youth to guide the development of tailored interventions, thereby supporting the clinical utility of the CHR-P concept.

**Supplementary Information:**

The online version contains supplementary material available at 10.1007/s00787-024-02491-x.

## Introduction

Three decades ago, specific criteria were developed to prospectively detect individuals with a substantial and putative risk of developing a full-blown psychotic disorder [1]. Since that time, specialized mental health clinics have been established to treat youths at clinical high risk for psychosis (CHR-P), with the aim of preventing, postponing, or ameliorating the transition to a full-blown psychotic disorder and improving general functioning [2–5]. However, crucial shortcomings challenge the relevance of the CHR-P paradigm in healthcare systems. First, only a small proportion (approximately one-third) of CHR-P individuals go on to develop a psychotic disorder [6]. Second, only a minority of first-episode psychosis cases (i.e., 4% in some research [7, 8]) are detected by at-risk mental state services prior to onset. Third, the CHR-P state may be challenging to differentiate from common mental disorders due to its clinical heterogeneity and the high prevalence of comorbid mental disorders [9,73, 74].

These shortcomings raise concerns about the diagnostic validity and clinical utility of the CHR-P concept, thereby questioning the need for specialized clinical centers for psychosis prevention, with implications for policymaking and the organization of health services. Indeed, a debate over the CHR-P paradigm has emerged, with critics questioning the concepts of “risk” and “transition” [10–12] and, more broadly, the CHR-P concept itself [7, 13]. Overall, this debate highlights the need for further research to clarify the diagnostic validity and clinical utility of the CHR-P concept.

First et al. [14] proposed formal definitions for the terms “diagnostic validity” and “clinical utility,” based on the functions or facets that characterize them. For example, the so-called “descriptive validity (i.e., whether the features of a category are unique to that category relative to other mental disorders)” refers to diagnostic validity, while the capability of “assisting clinicians in choosing effective interventions to improve clinical outcomes” refers to clinical utility ( [14], p. 947). Accordingly, it can be argued that the CHR-P concept is relevant if it denotes a specific (i.e., unique) clinical population and provides information that is useful for planning tailored interventions.

Innovative insights from the *network theory of mental disorders* [15–17] may further clarify the potential clinical specificities of CHR-P patients and identify relevant intervention targets to improve clinical outcomes—focusing on both the diagnostic validity and the clinical utility of the CHR-P concept. In fact, a growing body of research suggests that conceptualizing psychopathology in terms of dimensions, rather than categories, and adopting approaches aimed at comprehending complex system structures may improve our knowledge of mental disorders and early manifestations of psychopathology [18–21]. This conceptual framework posits that, rather than a “common cause” generating manifestations of psychopathology, it is the causal interplay among mutually reinforcing symptoms (i.e., symptoms that cause and reinforce each other) in a network structure (i.e., a web of associations among symptoms) that leads to what is phenomenologically identified as “mental disorder” [15, 22]. According to this perspective, each symptom may play a different role and hold different statistical power in maintaining and spreading manifestations of psychopathology [22, 23].

A network analysis might further clarify whether there are specific treatment targets that warrant a specialized clinical approach for CHR-P youths. In other words, exploring mutual interactions (i.e., conditional dependence) between clinical variables (rather than assessing the mere presence/absence or severity of specific symptoms) may help to confirm the clinical utility of the CHR-P concept (irrespective of transition) in clinical contexts.

The present study aimed at building and comparing three “multidimensional” network structures including subclinical positive, negative, disorganization, and general symptoms; depressive and anxiety symptoms; functioning; and an intelligence quotient (IQ). These structures were explored in samples of: (a) general help-seeking youth (both CHR-P and non–CHR-P) as encountered in clinical practice (i.e., *help-seekers network*); (b) CHR-P youth only (i.e., *CHR-P network*); and (c) non–CHR-P youth only (i.e., *non–CHR-P network*, in line with a recent review [24]). These variables were selected as they are clinically relevant in maintaining psychopathology manifestations in CHR-P youth [9, 25–28] and are widely assessed in daily clinical practice, promoting the reproducibility and generalizability of the study design and findings.

## Methods

### Participants and procedure

A national sample of 146 CHR-P and 103 non–CHR-P help-seeking children and adolescents was recruited consecutively from the Child and Adolescence Neuropsychiatric Unit of the “Bambino Gesù” Pediatric Hospital in Rome. This clinical service accepts referrals of young people who are suspected of being at risk of developing psychosis (which might result in *pre-assessment enrichment*, as observed in previous studies [29]), and provides preventive care to CHR-P individuals in an outpatient setting. The following inclusion criteria were applied: (a) under 25 years of age; (b) fluent in Italian; (c) no current or historical full-blown psychotic disorder, as assessed using the Schedule for Affective Disorders and Schizophrenia for School-Aged Children Present and Lifetime Version DSM-5 (K-SADS-PL DSM-5; [30]); (d) no organic syndrome, neurological disease, or brain injury that could complicate the assessment of the study variables; and (e) an IQ of 70 or above. The Structured Interview for Psychosis-Risk Syndromes (SIPS) (see “Measures” section) was administered to determine the presence/absence of the CHR-P condition, with assessments conducted by a licensed staff psychiatrist and a clinical psychologist. Participants were considered at CHR-P if they met at least one of the following SIPS inclusion criteria: attenuated psychotic symptoms (APS), brief intermittent psychotic syndrome (BIPS), and genetic risk deterioration syndrome (GRD), with no full-blown psychotic disorder and/or the presence of psychotic symptoms (POPS).

The research was approved by the Ethics Committee of the “Bambino Gesù” Pediatric Hospital (n◦2921/2022) and the Ethics Committee of the Department of Dynamic and Clinical Psychology and Health Studies, Sapienza University of Rome (n◦44/2017), and it was conducted in accordance with the 1964 Helsinki Declaration. Prior to engaging in the study, all participants and their parents (when the participant was a minor) provided written informed consent, indicating their understanding of the study procedures and their right to withdraw participation at any time, without penalty.

### Measures

Sociodemographic and baseline clinical characteristics (e.g., age and sex) were obtained from each patient’s clinical records. The following measures were employed in the study:

#### Structured interview for prodromal syndromes (SIPS) [27, 31]

This structured interview is designed to identify a CHR-P condition. It consists of four domains: (1) the Scale of Prodromal Symptoms (SOPS), (2) the DSM-IV Schizotypal Personality Disorder Checklist, (3) a questionnaire pertaining to family history of mental illness, and (4) the Global Assessment of Functioning scale. In the SIPS, 19 items examine the following clusters of symptoms: (a) positive symptoms, (b) negative symptoms, (c) disorganization symptoms, and (d) general symptoms. Each item is ranked from 0 (i.e., absence) to 6 (i.e., psychotic). If at least one item in the positive symptoms cluster receives a score of 3, 4, or 5, a CHR-P condition is assigned. Studies have reported a median agreement of kappa = 0.89 (range > 0.70–1.00) for the assignment of a CHR-P state, and a median reliability coefficient of 0.90 (range > 0.75–0.96) for the SIPS [32].

#### Children’s depression inventory (CDI) [33]

The CDI is a self-report measure that is used to assess depressive symptoms in children and adolescents aged 8–17 years. It is comprised of 27 items, each ranked 0, 1, or 2, providing a total score in the range of 0–54. Specific sets of items evaluate school and peer domains. Respondents answer items by reflecting on their feelings and thoughts over the past 2 weeks. In the present study, the CDI displayed high internal consistency (α = 0.80) [34].

#### Multidimensional anxiety scale for children (MASC) [35]

The MASC is a 39-item self-report questionnaire that is used to evaluate anxiety symptoms in children and adolescents. It contains four subscales: (1) physical symptoms, (2) social anxiety, (3) harm avoidance, and (4) separation anxiety. In the present study, the MASC displayed good internal consistency (α = 0.60 to α = 0.85) and high test–retest reliability (*r* = 0.79 to *r* = 0.93) [36, 37].

#### Global functioning: social (GF: social) and global functioning: role (GF: role) [38–40]

The measures provide two total scores in the range of 1–10 (with 10 indicating very high functioning and 1 indicating extreme dysfunction). For the present analyses, a mean score, considering the total scores for both GF-Social and GF-Role, was calculated and entered into the analysis as a global measure of overall psychological functioning. Both measures showed excellent inter-rater reliability and accuracy [43].

#### Wechsler intelligence scale for children – third edition and fourth edition (WISC-III; WISC-IV) and wechsler adult intelligence scale (WAIS-IV) [44–46]

The WISC-III, WIS-IV, and WAIS-IV are widely used instruments for assessing general intelligence. The WISC-III and WISC-IV are used with youth aged 4–16 years, while the WAIS-IV is used with adolescents and adults aged 16–90 years. The task scores of these measures derive a full-scale composite IQ score, which was used in the present study.

### Network estimation

We applied a network approach to investigate the interrelations among symptoms and other clinical variables. In a network structure, nodes represent clinical variables (e.g., symptoms, general functioning, IQ), while edges represent statistical relationships of dependence between two nodes. We selected specific study domains and summed up the relevant items, standardizing the resulting values. To estimate the network, we applied a Gaussian graphical model (GGM), using the measured correlations between variables as input data. The GGM uses pairwise correlations to infer the network structure [47, 48], showing the derived statistical relations between variables, as expressed by edge values. More precisely, the presence of an edge between two nodes indicates conditional dependence, and the edge value represents the strength of this dependence. Otherwise, if two nodes lack an edge, this suggests that the corresponding variables are conditionally independent, after accounting for all other variables in the network. The GGM is an undirected graphical model; hence, it does not indicate the direction of dependence between variables [47, 48]. While conditional dependence could reflect a direct causal relationship or be the effect of a latent variable (i.e., a common cause), the GGM does not provide this information. However, it can represent feedback loops.

In the network estimation, we used the LASSO operator (i.e., a regularization procedure) to shrink small edge values to 0, effectively forcing the model to drop weak edges from the network estimation. The tuning parameter was set to 0.5.

To analyze the resulting network structure, we calculated several centrality indices: *node strength* (i.e., the sum of the absolute values of all connection weights for a given node), *closeness* (i.e., the inverse sum of the shortest paths between a given node and all other nodes), *betweenness* (i.e., the proportion of shortest paths between any two nodes passing through a given node), and *expected influence* (i.e., the sum of the connection weights for a given node, including their signs) [49–51]. To measure the stability of these centrality indices, we calculated the correlation stability coefficient (CS). CS represents the maximum proportion of the population that can be dropped while maintaining a recalculated index correlation of at least 0.7 with the indices of the complete original sample. A CS value of 0.25 is considered acceptable, 0.5 is considered good, and 0.70 and above is considered excellent. The network analysis was performed following the relevant indications in the literature [15, 48, 49].

We conducted a network analysis on the sample of help-seeking children and adolescents (both CHR-P and non–CHR-P) and other network analyses on the CHR-P and non-CHR-P subsamples. The analyses were performed using the R statistical program (version 4.2.2; R_Core_Team, 2013), employing the *qgraph* and *bootnet* packages.

## Results

### Sample characteristics

An initial sample of *N* = 291 met the study criteria. However, 42 CHR-P youth (15.5%) were excluded due to missing data, in line with previous studies [21, 52]. Table S2 provides the characteristics of the included and excluded participants. The final sample comprised 249 participants, of whom 146 (58.6%) met the criteria for a CHR-P condition. Table 1 and Table S1 report the demographic information and comorbid diagnoses for both samples. The mean age of CHR-P patients was 14.31 years (*SD* = 2.09), while the mean age of non–CHR-P patients was 12.58 years (*SD* = 2.46).

**Table 1:** Demographic characteristics of CHR-P and non–CHR-P patients

	CHR-P (*N* = 146)	Non–CHR-P (*N* = 103)	
Age, years (*M*, *SD*)	14.31 (2.09)	12.58 (2.46)	
Sex (m) %	70 (47.95)	80 (77.67)	
Global functioning (*M*, *SD*)	4.16 (0.95)	4.85 (0.71)	
Positive symptoms (*M*, *SD*)	11.36 (3.53)	1.51 (2.31)	
Negative symptoms (*M*, *SD*)	17.64 (7.42)	8.16 (0.50)	
Disorganization symptoms (*M*, *SD*)	9.88 (4.64)	4.25 (0.86)	
General symptoms (*M*, *SD*)	11.27 (4.81)	5.54 (0.78)	
IQ (*M*, *SD*)	97.98 (14.09)	95.16 (11.30)	
Anxiety symptoms (*M*, *SD*)	59.95 (14.32)	50.94 (10.78)	
Depressive symptoms (*M*, *SD*)	17.28 (10.32)	11.33 (5.73)	

### Network analysis

**Fig. 1:**
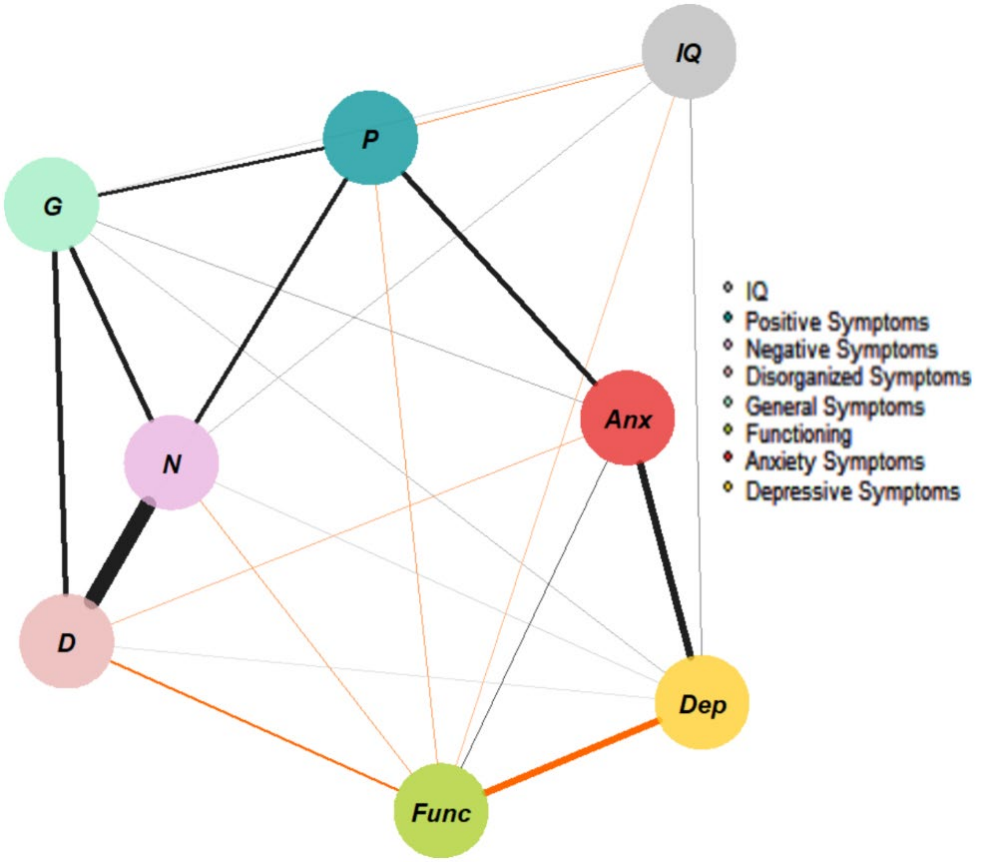
Network structure of help-seeking individuals (assessed using the SIPS, CDI, MASC, GF, and WISC/WAIS). The associations are either positive (colored black) or negative (colored red), with thicker lines representing stronger associations

**Fig. 2:**
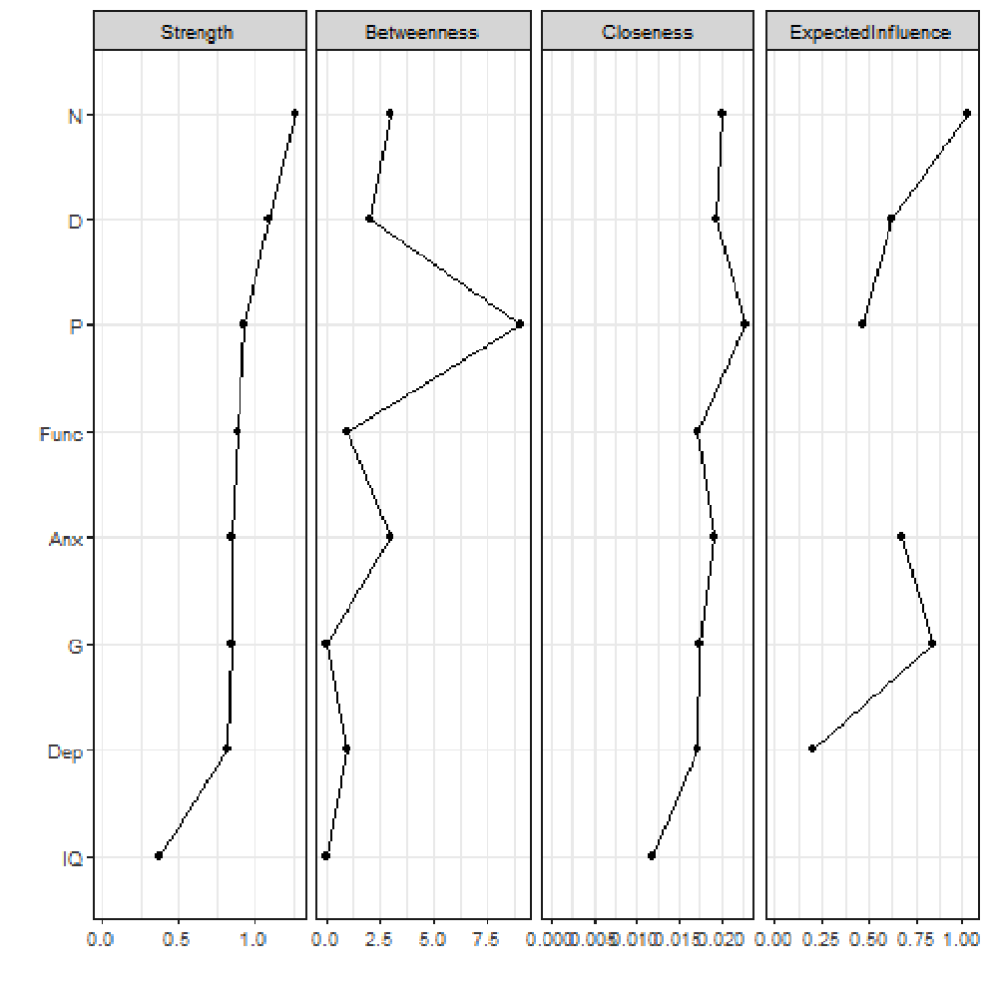
Centrality indices of the study variables within the network of help-seeking individuals. Note: centrality indices (i.e., node strength, closeness, betweenness, expected influence) are shown as standardized *z*-scores

Figure 1 shows the network estimated from the entire sample (i.e., both CHR-P and non–CHR-P patients). The highest node strength was observed for *negative symptoms*. Visual inspection revealed that each node in the network presented at least one edge. *Positive symptoms* were positively connected to *negative*, *general*, and *anxiety symptoms*, and negatively connected to *functioning* and *IQ*. Figure S1 displays the correlation matrix, and Fig. 2 plots the network centrality indices. The network CS was 0.75, indicating excellent stability properties (see supplementary materials, Figure S2). The bootstrapped confidence intervals for the estimated edge weights are reported in the supplementary materials (Figure S3).

**Fig. 3:**
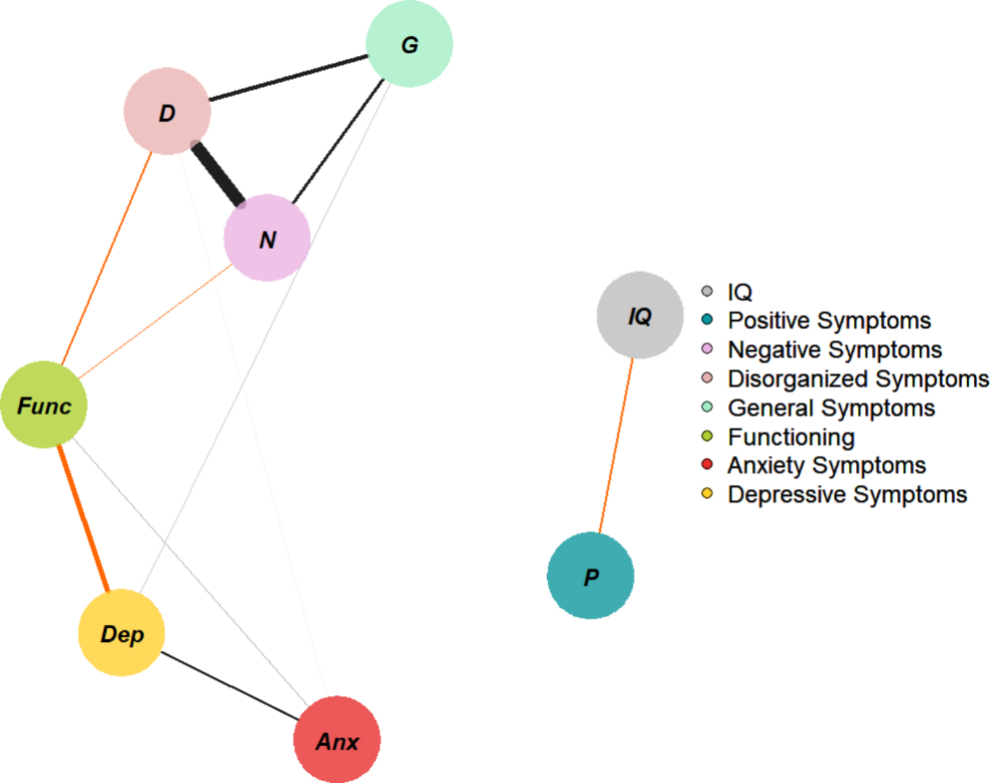
Network structure of CHR-P individuals (assessed using the SIPS, CDI, MASC, GF, and WISC/WAIS). The associations are either positive (colored black) or negative (colored red), with thicker lines representing stronger associations

**Fig. 4:**
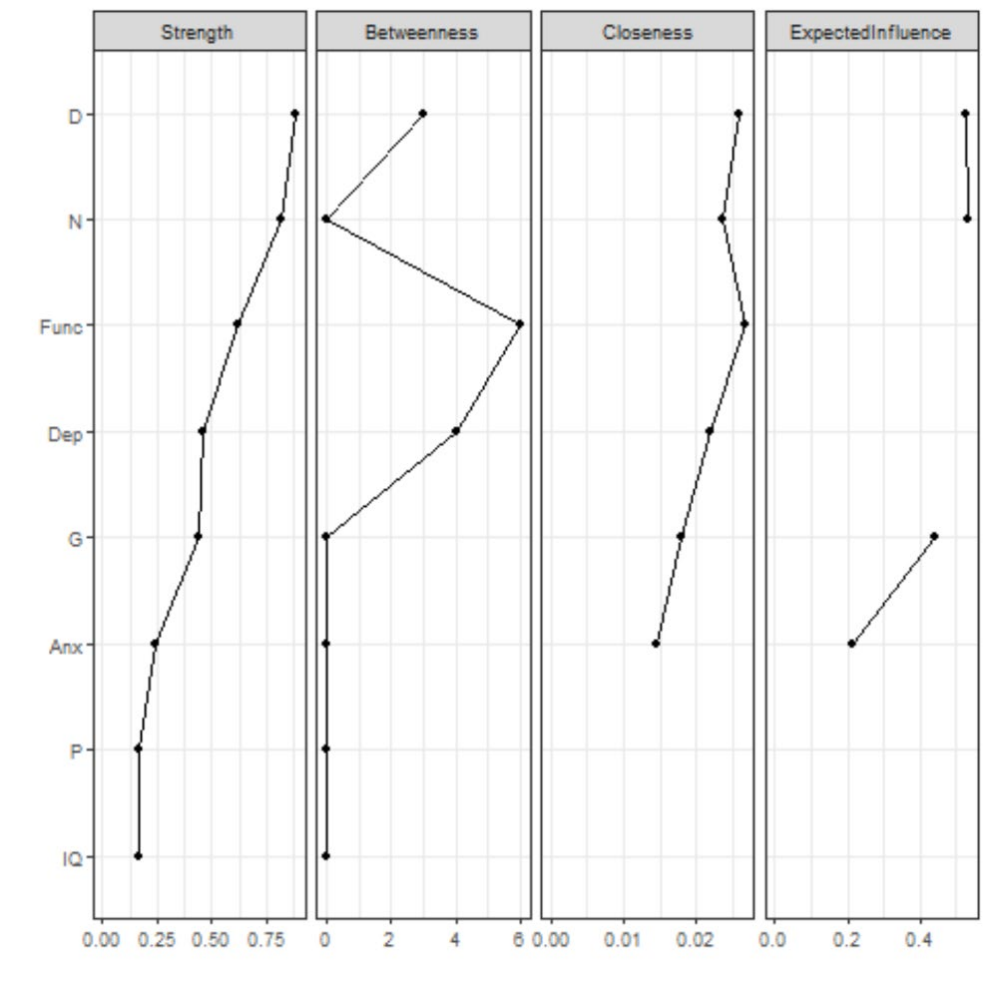
Centrality indices of the study variables within the network of CHR-P individuals. Note: centrality indices (i.e., node strength, closeness, betweenness, expected influence) are shown as standardized *z*-scores

Figure 3 shows the network with only CHR-P participants. The highest node strength was observed for *disorganization symptoms*. Visual inspection revealed two separate “archipelagos of symptoms”: (i) a subgraph consisting of *anxiety* and *depressive symptoms; functioning*; and *negative, disorganization, and general symptoms*; and (ii) a subgraph consisting of only *IQ* and *positive symptoms*. *Functioning* was negatively connected to *depressive, negative, and disorganization symptoms*, and positively connected to *anxiety symptoms*. Figure S4 presents the correlation matrix. *Positive symptoms* displayed only a negative connection with *IQ*. Figure 4 plots the network centrality indices. The CS for node strength was 0.51, indicating good stability properties (see supplementary materials, Figure S5). The bootstrapped confidence intervals for the estimated edge weights are reported in the supplementary materials (Figure S6).

**Fig. 5:**
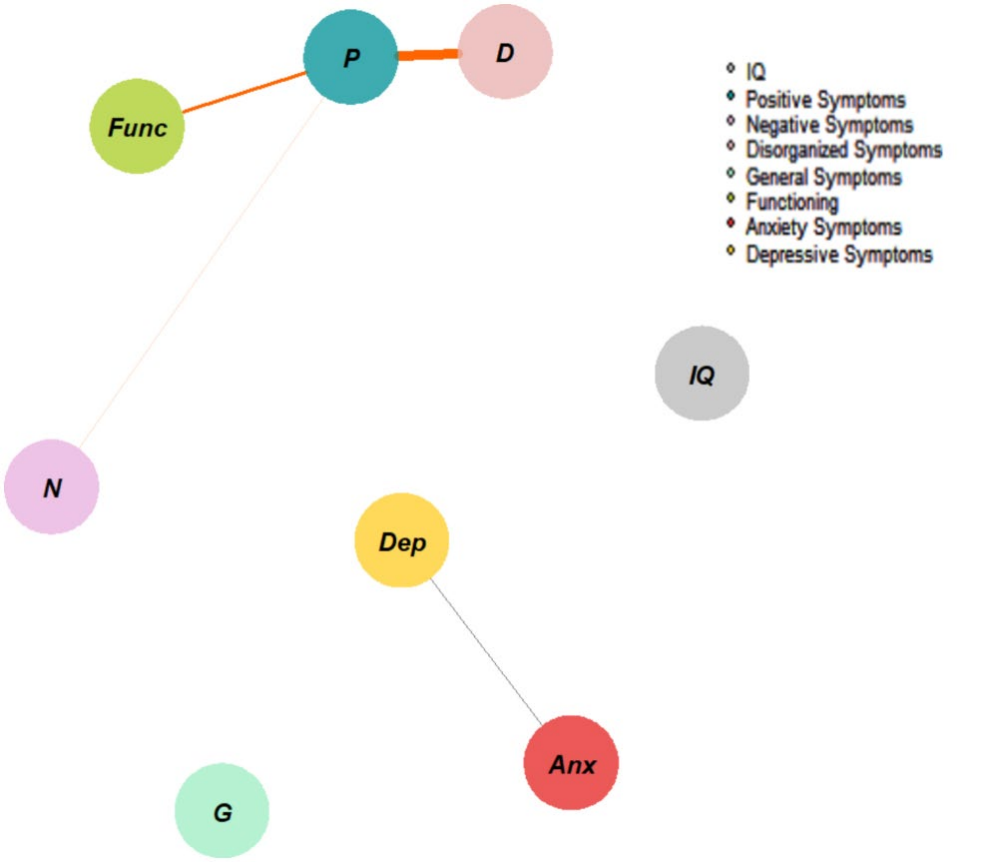
Network structure of non–CHR-P individuals (assessed using the SIPS, CDI, MASC, GF, and WISC/WAIS). The associations are either positive (colored black) or negative (colored red), with thicker lines representing stronger associations

**Fig. 6:**
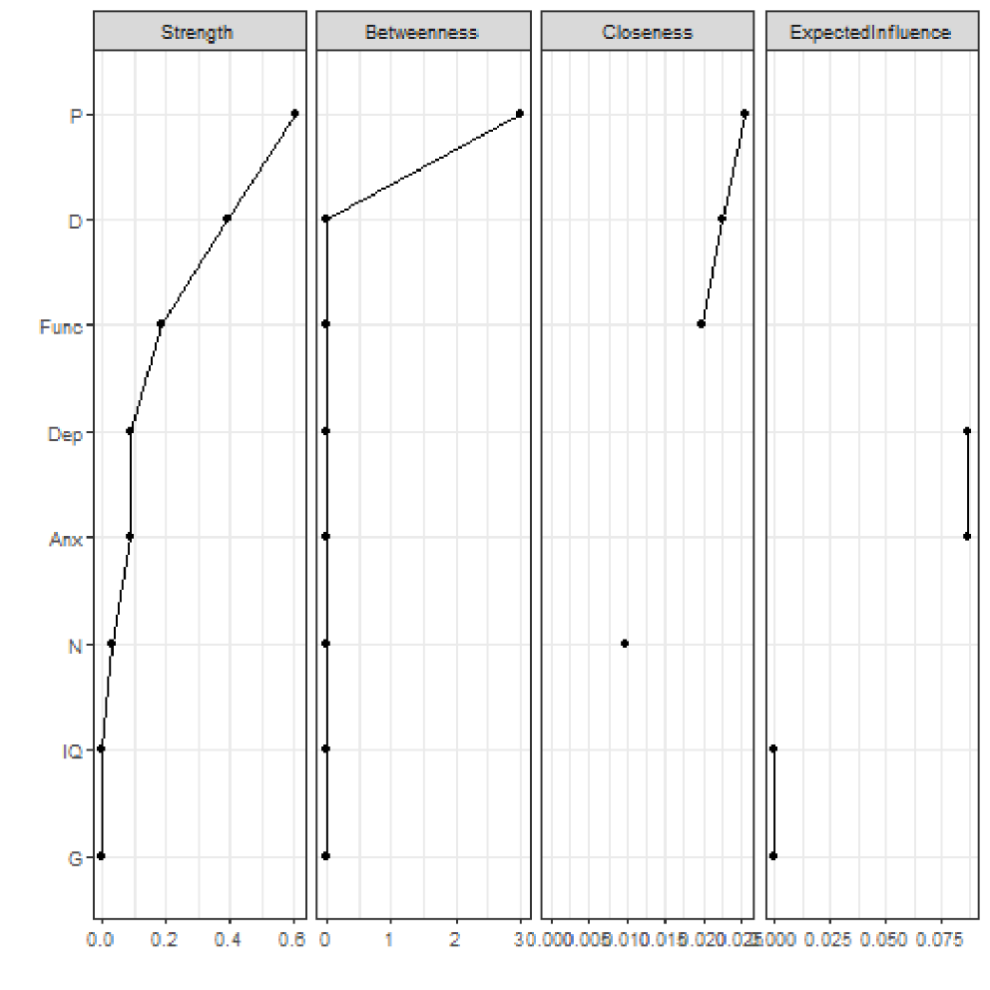
Centrality indices of the study variables within the network of non–CHR-P individuals. Note: centrality indices (i.e., node strength, closeness, betweenness, expected influence) are shown as standardized *z*-scores

Figure 5 shows the network with only non–CHR-P participants. The highest node strength was observed for *positive* symptoms, which were negatively connected to *disorganization* and *negative symptoms*, as well as *functioning*. *Depressive symptoms* were positively connected to *anxiety symptoms*. Figure 6 plots the network centrality indices, and Figure S7 presents the correlation matrix. The CS for node strength was 0.28, indicating acceptable stability properties (see supplementary materials, Figure S8). The bootstrapped confidence intervals for the estimated edge weights are reported in the supplementary materials (Figure S9).

## Discussion

The present study aimed at applying a network analysis to investigate similarities and differences between a generic help-seeking youth population (CHR-P) and non–CHR-P youth. The primary goal was to identify the clinical specificities of CHR-P youth.

The first network structure, estimated from the entire help-seeking sample (including both CHR-P and non–CHR-P individuals, as they consecutively appeared in the clinical service), revealed significant interrelations among the included variables. Specifically, all variables (i.e., subclinical positive, negative, disorganization, and general symptoms; depressive and anxiety symptoms; functioning; IQ) were directly or indirectly connected to all others. According to the network theory of mental disorders, this interconnected configuration (with no subgraphs present) suggests that if a triggering factor such as a challenging life event activates one node, the activation may spread easily throughout the network, potentially sustaining the psychopathological manifestation [15, 22]. Accordingly, this clinical sample may benefit from interventions targeting any symptom in the network, since all nodes are interconnected. However, further longitudinal network analyses are required to validate this hypothesis [53].

Notably, different network architectures emerged when the network analysis was applied exclusively to CHR-P and non–CHR-P samples. In the CHR-P network, in contrast to the general help-seekers network, we observed two distinct subgraphs (i.e., “archipelagos of symptoms”). The first subgraph included depressive and anxiety symptoms; negative, disorganization, and general symptoms; and functioning. The second subgraph consisted of positive symptoms and IQ. This network structure differed from that of the non–CHR-P sample, which showed only negative connections between positive symptoms and other nodes (i.e., disorganization and negative symptoms, general functioning) and a positive connection between depressive and anxiety symptoms.

In all three networks, depressive symptoms showed a positive connection with anxiety symptoms, suggesting that non-psychotic symptomatology may play a critical role in maintaining the network’s “state of harmful equilibrium” [22]. For example, in CHR-P youth, more severe depressive symptoms could lead to both more severe anxiety symptoms and a decline in functioning. Further research is needed to test this hypothesis, which, following the network approach [15, 53], is intrinsically bi-directional (i.e., poorer functioning or more severe anxiety could also impact depressive symptomatology). Notably, non-psychotic symptoms may represent the primary motivation for individuals to seek help in a specialized center^75^, ^76^, alongside factors such as mental health literacy [54, 55], regardless of the simultaneous presence of subclinical psychotic symptoms [56]. Moreover, recent longitudinal evidence has provided support for the impact of non-psychotic symptoms on psychopathology manifestations in CHR-P individuals [57]. However, according to our results, treating CHR-P youth by targeting depressive and anxiety symptoms may not affect positive symptoms, as these belong to a separate subgraph in the network.

In the CHR-P network, functioning bridged the gap between negative and depressive symptoms. This result aligns with meta-analytical evidence showing an association between depressive symptoms and impaired global functioning [58], as well as a relationship between negative symptoms (e.g., anhedonia, avolition, blunted affect) and poor social and role functioning [59]. An explanatory hypothesis for the bridging role played by functioning may lie in the impact of negative symptoms, which hinder active participation in several areas of life [60], potentially triggering symptoms of depression. However, also in this case, the relationship is bi-directional (i.e., depression may trigger poor functioning, which in turn may activate negative and disorganization symptoms).

Positive symptoms played different roles across the three analyzed network structures, thereby representing the main point of difference across the networks. In the first network (i.e., help-seeking subjects), positive symptoms were connected to comorbid symptoms and functioning. However, in the CHR-P network, positive symptoms were isolated, connected only to IQ. Finally, in the non–CHR-P network, they were negatively connected to negative and disorganization symptoms, as well as functioning. This suggests that positive symptoms in CHR-P individuals may require specific treatment strategies, as they may not interact with other psychopathology domains [61]. In fact, the “isolation” of positive symptoms may be a defining feature of CHR-P status, possibly developing over time. This observation is consistent with previous findings from our research group showing that positive symptoms do not always display meaningful connections with other nodes in the network, marking them as potential evaluation and intervention targets [21]. A similar tendency for psychotic symptoms to cluster separately from non-psychotic dimensions (e.g., risk of developing depressive, bipolar, or borderline personality disorder) has been observed in recent research adopting a transdiagnostic approach [62].

IQ also functioned differently across the three networks. In the help-seekers network, it showed connections with multiple nodes, while in the non–CHR-P network, it had no connections, and in the CHR-P network, it connected with only positive symptoms. This variation underscores the importance of assessing cognitive domains in CHR-P individuals [28]. Finally, while anxiety symptoms and functioning exhibited a positive correlation in the CHR-P sample, they were not significantly related in the broader help-seeking sample or the non–CHR-P sample.

The abovementioned critical differences between the three network structures suggest varying clinical needs that must be addressed in clinical practice, consistent with other reports from mental disorder prevention services in Italy, indicating a general need for mental healthcare [63]. In other words, our results indicate that CHR-P youths may require specific clinical strategies, even if their risk of transitioning to psychosis is unclear. This supports the clinical utility of psychosis at-risk diagnostic concepts, as well as the specialized treatments offered in clinics for early detection and intervention.

Overall, the results support the diagnostic validity of the CHR-P concept, as the CHR-P network displayed specific (i.e., unique) interconnections that were not observed in the help-seekers and non–CHR-P networks. Moreover, our findings also indicate that tailored intervention strategies are needed to improve the clinical outcomes for CHR-P individuals, reinforcing the clinical utility of the CHR-P concept. Finally, the results suggest that the CHR-P state may be better conceptualized as a system with complex interactions among clinical variables, rather than a single syndrome [64].

Despite these promising findings and their implications for future research, the present study suffered from some limitations. First, the CHR-P individuals were younger (mean age = 14.32 years) compared to the CHR-P populations enrolled in most studies (mean age = 20.6 years [65]). Some scholars have questioned the predictive value of CHR-P criteria in younger populations [66, 67]. While this is not necessarily a study limitation, it highlights the need for further research into the unique developmental characteristics of younger CHR-P individuals. Such research could contribute to the development of diagnostic tools and intervention strategies for these individuals [68, 69], who are characterized by a higher prevalence of unusual perceptual experiences and attenuated hallucinations compared to older patients [70]. Second, a proportion of the initial sample (i.e., approximately 15% of the CHR-P youth) was excluded due to missing data. Nevertheless, the subsample including missing data showed minimal differences compared to the included group (see also Table S2). Third, the study did not provide evidence regarding the interactions between pairs of symptoms over time, indicating a need for future longitudinal studies to better understand these relationships. Fourth, biological markers were not considered in the study, despite their significant relevance to CHR-P children and adolescents [68, 69]. Fifth, the generalizability of the results is limited to a single clinical service in a Western country. Finally, “basic symptoms” were not taken into account. Since these refer to a set of criteria reflecting an earlier phase of risk than the at-risk condition considered in this network analysis [71], they should be included in future network studies. Nevertheless, the current study explored the specific characteristics of a “late” prodromal phase indicating a more proximal risk for developing psychosis, constituting the primary focus of CHR-P assessment and intervention [3, 65, 72].

## Electronic supplementary material

Below is the link to the electronic supplementary material.

Supplementary Material 1

## Data Availability

The data that support the findings of this study are available from the corresponding author upon reasonable request.
