# Supplementary material for: Clinical utility of the at-risk for psychosis state beyond transition: A multidimensional network analysis
Source: Eur Child Adolesc Psychiatry. 2024 Jun 19;34(1):287–96. doi: 10.1007/s00787-024-02491-x (PMC11805795; doi:10.1007/s00787-024-02491-x)
Supplement: Supplementary file 1 — Supplementary Material 1 [file 787_2024_2491_MOESM1_ESM.docx]

**Journal:** European Child & Adolescent Psychiatry

**Supplementary materials to**

**Title**: Clinical utility of the at-risk for psychosis state beyond transition: A multidimensional network analysis

**Authors:** Tommaso Boldrini^1^, Gabriele Lo Buglio^2,*^, Erika Cerasti^2^, Maria Pontillo^3^, Laura Muzi^4^, Silvia Salcuni^5^, Andrea Polari^6,7^, Stefano Vicari^3,8^, Vittorio Lingiardi^2^, Marco Solmi^9,10,11,12,13^

**Affiliations:**

1. Department of Psychology and Educational Science, Pegaso Telematic University, Naples, Italy
2. Department of Dynamic and Clinical Psychology, and Health Studies, Faculty of Medicine
   and Psychology, Sapienza University of Rome, Rome, Italy
3. Child Psychiatry Unit, Department of Neuroscience Bambino Gesù Children’s Hospital, IRCCS, Rome, Italy
4. Department of Philosophy, Social Sciences, Humanities and Education, University of Perugia, Perugia, Italy
5. Department of Developmental Psychology and Socialization, University of Padua, Padua, Italy
6. Orygen Specialist Programs, Melbourne, Australia
7. Centre for Youth Mental Health, University of Melbourne, Australia
8. Department of Life Science and Public Health, Catholic University of the Sacred Heart, Rome, Italy
9. School of Epidemiology and Public Health, Faculty of Medicine, University of Ottawa, Ottawa, ON, Canada
10. Department of Psychiatry, University of Ottawa, Ottawa, ON, Canada
11. On Track: The Champlain First Episode Psychosis Program, Department of Mental Health, The Ottawa Hospital, Ontario, Canada.
12. Ottawa Hospital Research Institute (OHRI) Clinical Epidemiology Program University of Ottawa, Ottawa, ON, Canada
13. Department of Child and Adolescent Psychiatry, Charité Universitätsmedizin, Berlin, Germany

*Corresponding author

Gabriele Lo Buglio

Department of Dynamic and Clinical Psychology, and Health Studies, Faculty of Medicine
and Psychology, Sapienza University of Rome, Rome, Italy. gabriele.lobuglio@uniroma1.it

**Index**

**Table S1.** Comorbid Axis I diagnoses (including double diagnoses) of CHR-P and non–CHR-P patients.

**Table S2.** Comparisons between CHR-P individuals included in the study and those excluded due to missing data.

**Figure S1.** Correlation matrix for help-seeking individuals.

**Figure S2.** Average correlation between the centrality indices of the network subsamples and the original sample in the help-seeking network.

**Figure S3.** Bootstrapped confidence intervals of estimated edge weights for the help-seeking network.

**Figure S4.** Correlation matrix for CHR-P individuals.

**Figure S5.** Average correlation between the centrality indices of the network subsamples and the original sample in the CHR-P network.

**Figure S6.** Bootstrapped confidence intervals of estimated edge weights for the CHR-P network.

**Figure S7.** Correlation matrix for non–CHR-P individuals.

**Figure S8.** Average correlation between the centrality indices of the network subsamples and the original sample in the non–CHR-P network.

**Figure S9.** Bootstrapped confidence intervals of estimated edge weights for the non–CHR-P network.

**Table S1.** Comorbid Axis I diagnoses (including double diagnoses) of CHR-P and non-CHR-P patients

| Comorbid diagnosis, *n* % |  |  |
| --- | --- | --- |
|  | CHR-P patients (*N*=146) | Non–CHR-P patients (*N*=103) |
| Depressive disorders | 70 (47.95) | 26 (25.24) |
| Obsessive-compulsive and related disorders | 45 (30.82) | 14 (13.59) |
| Anxiety disorders | 34 (23.29) | 26 (25.24) |
| Disruptive, impulse-control, and conduct disorders | 6 (4.11) | 16 (15.53) |
| Feeding and eating disorders | 5 (3.43) | 1 (0.97) |
| Neurodevelopmental disorders | 11 (7.53) | 5 (4.85) |
| Bipolar and related disorders | 4 (2.74) | 1 (0.97) |
| Trauma- and stressor-related disorders | 2 (1.37) | 3 (2.91) |
| Elimination disorders  No mental disorder | 1 (0.69)  16 (10.46) | 1 (0.97)  18 (17.48) |

Legend: CHR-P refers to clinical high risk for psychosis.

**Table S2.** Comparisons between CHR-P individuals included in the study and those excluded due to missing data.

|  |  |  |  | | | | | | | | | | | | | | | | | | |
| --- | --- | --- | --- | --- | --- | --- | --- | --- | --- | --- | --- | --- | --- | --- | --- | --- | --- | --- | --- | --- | --- |
|  | | | | | **Group** | | ***N*** | | **Mean/Total** | | ***SD*** | |  | |  | |  | |  | |  |
| Age | | | |  | 1 |  | 146 |  | 14.32 |  | 2.09 |  | |  | | *t*=1.254 | |  | |  |  |
|  | | | | | 2 |  | 42 |  | 13.83 |  | 2.537 |  | |  | |  | |  | |  |  |
| Intelligence quotient | | | |  | 1 |  | 146 |  | 97.98 |  | 14.09 |  | |  | | *t*=0.722 | |  | |  |  |
|  | | | | | 2 |  | 42 |  | 97.10 |  | 14.405 |  | |  | |  | |  | |  |  |
| Sex (M) | | | | | 1 |  | 146 |  | 70 |  |  |  | |  | | x^2^=9.41** | |  | |  |  |
|  | | | | | 2 |  | 42 |  | 9 |  |  |  | |  | |  | |  | |  |  |
| Anxiety symptoms | | | |  | 1 |  | 146 |  | 59.95 |  | 14.32 |  | |  | | *t*=0.431 | |  | |  |  |
|  | | | | | 2 |  | 3 |  | 56.33 |  | 18.771 |  | |  | |  | |  | |  |  |
| Depressive symptoms | | | |  | 1 |  | 146 |  | 17.28 |  | 10.32 |  | |  | | *t*=0.224 | |  | |  |  |
|  | | | | | 2 |  | 7 |  | 12.43 |  | 8.696 |  | |  | |  | |  | |  |  |
| Global functioning | | | |  | 1 |  | 146 |  | 4.38 |  | 1.16 |  | |  | | *t*=2.299* | |  | |  |  |
|  | | | | | 2 |  | 42 |  | 3.95 |  | 0.539 |  | |  | |  | |  | |  |  |
| Positive symptoms | | | |  | 1 |  | 146 |  | 11.36 |  | 3.53 |  | |  | | *t*=0.610 | |  | |  |  |
|  | | | | | 2 |  | 42 |  | 10.98 |  | 3.904 |  | |  | |  | |  | |  |  |
| Negative symptoms | | | |  | 1 |  | 146 |  | 17.64 |  | 7.42 |  | |  | | *t*=-0.752 | |  | |  |  |
|  | | | | | 2 |  | 40 |  | 18.68 |  | 8.824 |  | |  | |  | |  | |  |  |
| Disorganization symptoms | | | |  | 1 |  | 146 |  | 9.88 |  | 4.64 |  | |  | | *t*=-1.490 | |  | |  |  |
|  | | | | | 2 |  | 42 |  | 11.10 |  | 4.648 |  | |  | |  | |  | |  |  |
| General symptoms | | | |  | 1 |  | 146 |  | 11.27 |  | 4.81 |  | |  | | *t*=0.519 | |  | |  |  |
|  | | | | | 2 |  | 40 |  | 10.82 |  | 4.640 |  | |  | |  | |  | |  |  |
|  |  |  |  | | | | | | | | | | | | | | | | | | |

 **p* ≤ .05; ***p* ≤ .01; 1 = included; 2 = excluded.


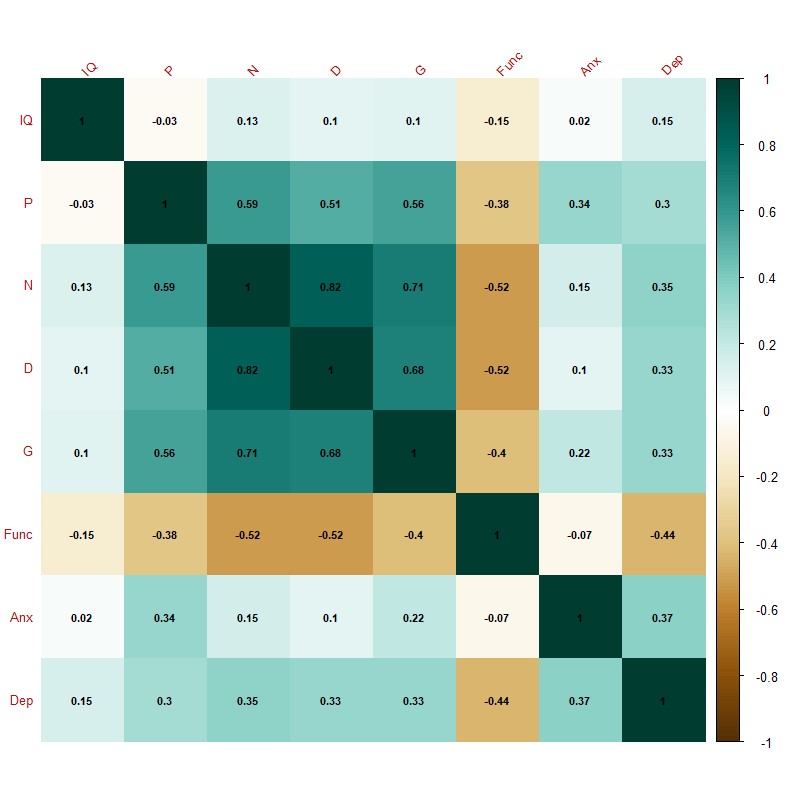


**Figure S1.** Correlation matrix for help-seeking individuals. Anx = anxiety symptoms; D = disorganization symptoms; Dep = depressive symptoms; Func = functioning; G = general symptoms; IQ = intelligence quotient; N = negative symptoms; P = positive symptoms.


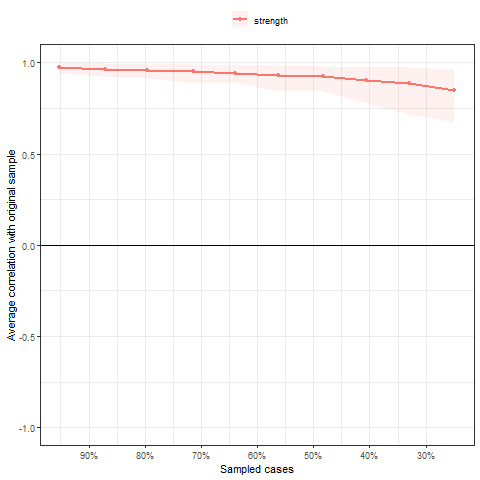


**Figure S2.** Average correlation between the centrality indices of the network subsamples and the original sample in the help-seeking network. Lines indicate means and areas indicate the range from the 2.5th to the 97.5th quantile.


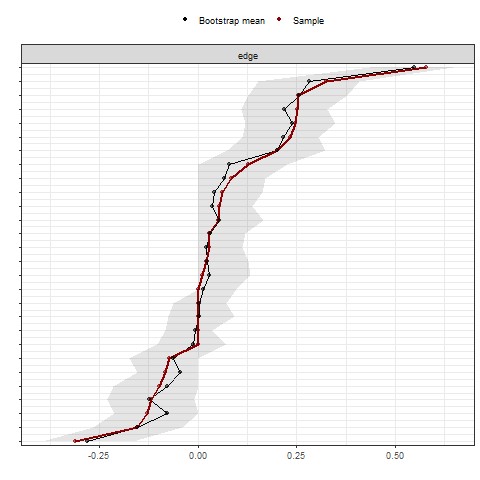


**Figure S3.** Bootstrapped confidence intervals of estimated edge weights for the help-seeking network.


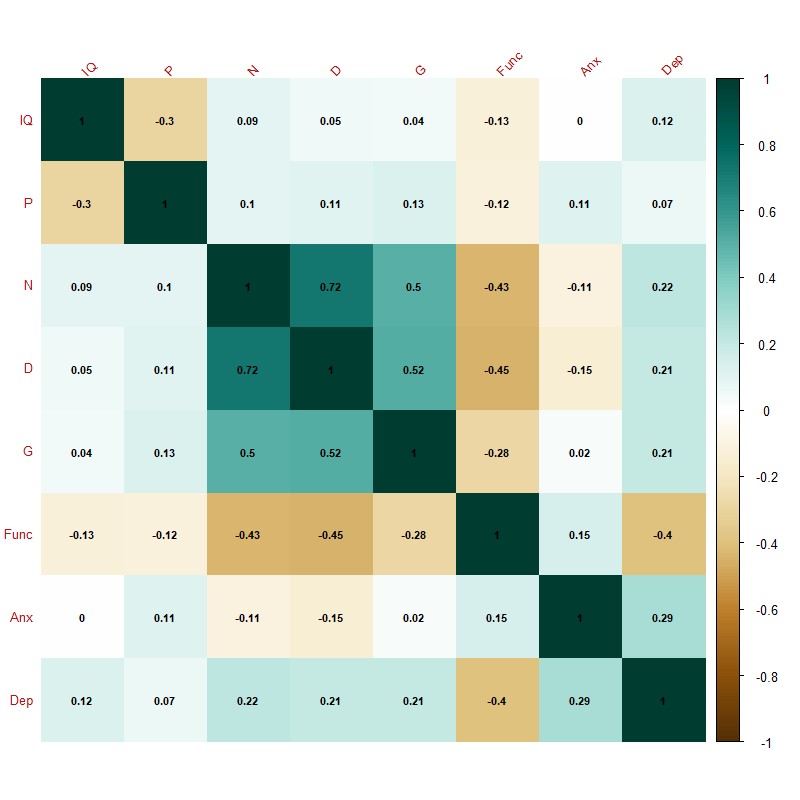


**Figure S4.** Correlation matrix for CHR-P individuals. Anx = anxiety symptoms; D = disorganization symptoms; Dep = depressive symptoms; Func = functioning; G = general symptoms; IQ = intelligence quotient; N = negative symptoms; P = positive symptoms.


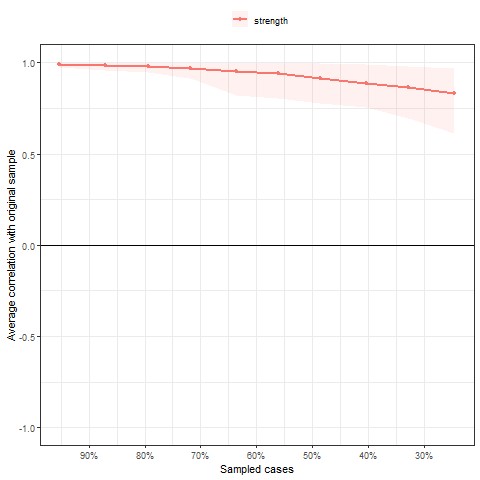


**Figure S5.** Average correlation between the centrality indices of the network subsamples and the original sample in the CHR-P network. Lines indicate means and areas indicate the range from the 2.5th to the 97.5th quantile.


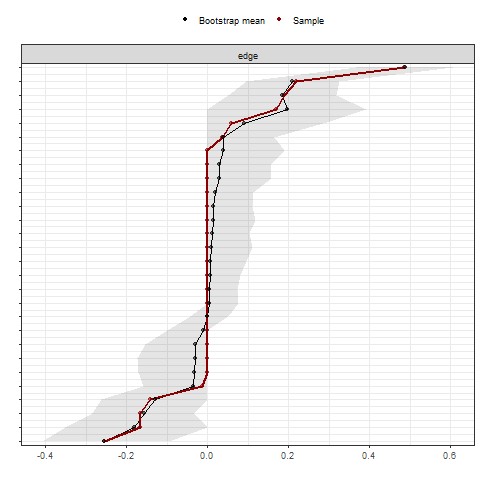


**Figure S6.** Bootstrapped confidence intervals of estimated edge weights for the CHR-P network.


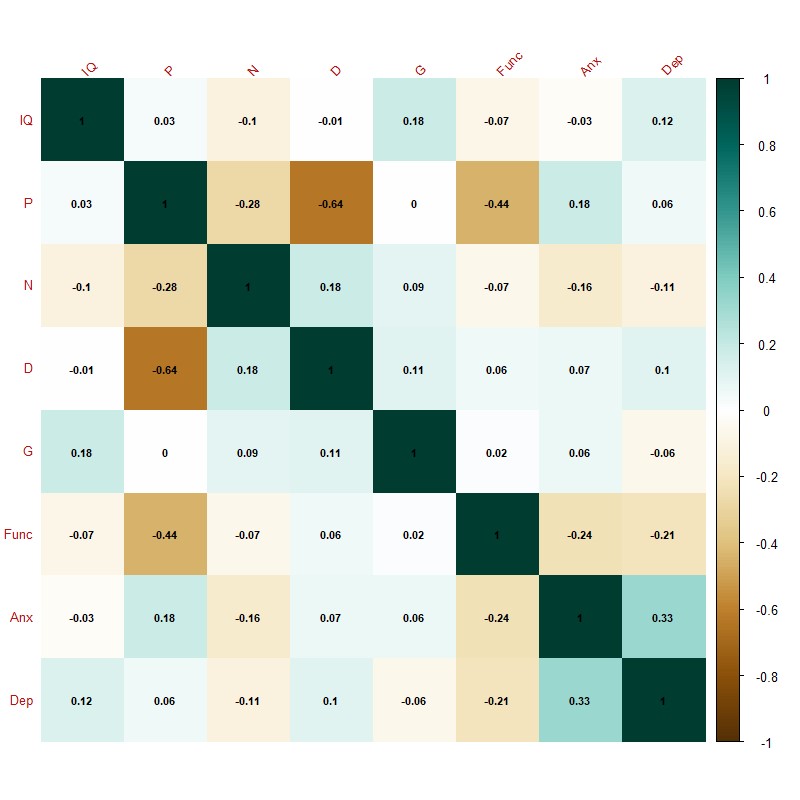


**Figure S7.** Correlation matrix for non–CHR-P individuals. Anx = anxiety symptoms; D = disorganization symptoms; Dep = depressive symptoms; Func = functioning; G = general symptoms; IQ = intelligence quotient; N = negative symptoms; P = positive symptoms.


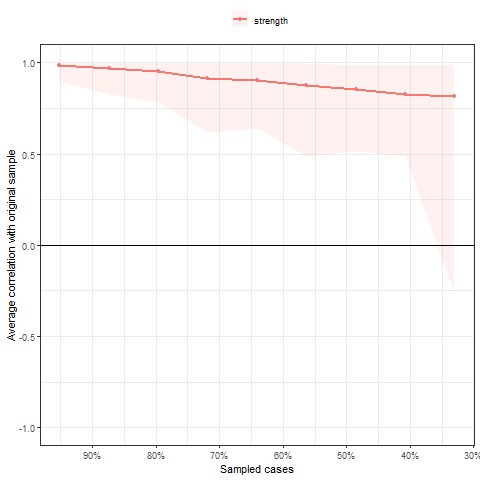


**Figure S8.** Average correlation between the centrality indices of the network subsamples and the original sample in the non–CHR-P network. Lines indicate means and areas indicate the range from the 2.5th to the 97.5th quantile.


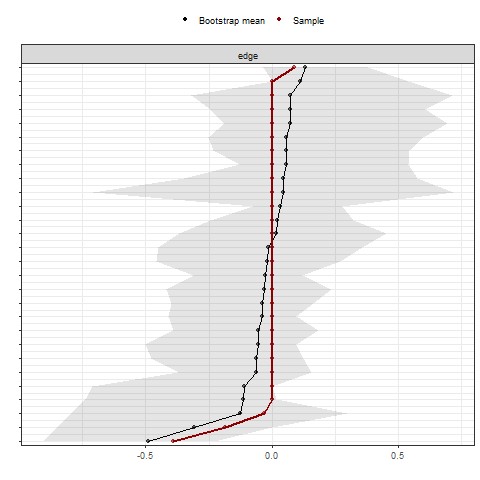


**Figure S9.** Bootstrapped confidence intervals of estimated edge weights for the non–CHR-P network.
